# E2F-1 targets miR-519d to regulate the expression of the ras homolog gene family member C

**DOI:** 10.18632/oncotarget.14833

**Published:** 2017-01-27

**Authors:** Xiu-Bo Sang, Zhi-Hong Zong, Li-Li Wang, Dan-Dan Wu, Shuo Chen, Bo-Liang Liu, Yang Zhao

**Affiliations:** ^1^ Department of Gynecology, The First Affiliated Hospital of China Medical University, Shenyang 110001, P. R. China; ^2^ Department of Biochemistry and Molecular Biology, College of Basic Medicine, China Medical University, Shenyang 110001, P. R. China

**Keywords:** ovarian carcinoma, E2F1, microRNA-519d, RhoC, tumorigenesis and progression

## Abstract

*E2F1* (E2F transcription factor 1) can act as a tumor suppressor or oncogene. We report the molecular mechanism of E2F1 in ovarian carcinoma tumorigenesis and progression. E2F1 expression levels in ovarian carcinoma tissue were examined by immunohistochemistry. After E2F1 plasmid transfection and E2F1-microRNA-519d (miR-519d)/si-RhoC (Ras homolog gene family member C) co-transfection, ovarian cancer cell phenotypes and the related molecules were examined *in vitro* and *in vivo*. *E2F1* was overexpressed in type I and type II ovarian carcinoma as compared to normal ovary tissues and normal fallopian tube tissues, respectively. *E2F1* overexpression promoted cell proliferation, G1–S progression, survival, migration, and invasion *in vitro*; miR-519d or siRhoC co-transfection reversed E2F1 oncogenic effects. *E2F1* overexpression promoted tumor growth *in vivo*; miR-519d overexpression inhibited it. *E2F1* overexpression increased RhoC, Bcl-2, cyclin D1, survivin, MMP2 (matrix metalloproteinase 2), MMP9, STAT3 (signal transducer and activator of transcription 3), and HuR (ELAV-like RNA-binding protein 1) expression; miR-519d overexpression decreased their expression. E2F1 downregulated miR-519d directly and miR-519d downregulated RhoC directly. Conversely, miR-519d directly downregulated E2F1, There is a direct repressive regulatory loop between E2F1 and miR-519d. We provide evidence that E2F1/miR-519d/RhoC is a promising signaling pathway for diagnosing and treating ovarian carcinoma.

## INTRODUCTION

Epithelial ovarian carcinoma is the most lethal gynecological malignancy, and has consistently low 5-year relative survival rates [1–5]. Difficult early diagnosis and frequent recurrence and metastasis lead to the high mortality rate of epithelial ovarian carcinoma. Exploring the underlying molecular mechanisms of epithelial ovarian carcinoma is essential for developing effective diagnostic and treatment strategies.

E2F transcription factor family members regulate the cell cycle, proliferation, apoptosis, and differentiation. Aberrant *E2F* gene expression is frequently documented in many cancers [6–10]. Among them, *E2F1* (E2F transcription factor 1) has drawn much attention because of its complex and diverse functions in different cancers [11–24]. E2F1 suppresses gastric cancer by downregulating Bcl-2, cyclin D1, and survivin [17], and inhibits Hodgkin lymphoma by upregulating p53 expression [25]. Conversely, it plays an oncogenic role by enhancing matrix metalloproteinase 9 (MMP9) transcription during invasion and metastasis in small cell lung cancer [28] and by activating MMP2 and MMP9 in local and vascular infiltration in clear cell renal cell carcinoma [29]. E2F1 expression is increased in ovarian carcinoma tissues, and it is believed that E2F1 is a promoter of the development of ovarian carcinoma [26–30]. However, the exact underlying molecular mechanism remains elusive.

## RESULTS

### The correlation between E2F1 expression and ovarian carcinoma pathogenesis and aggressiveness

**Figure 1:**
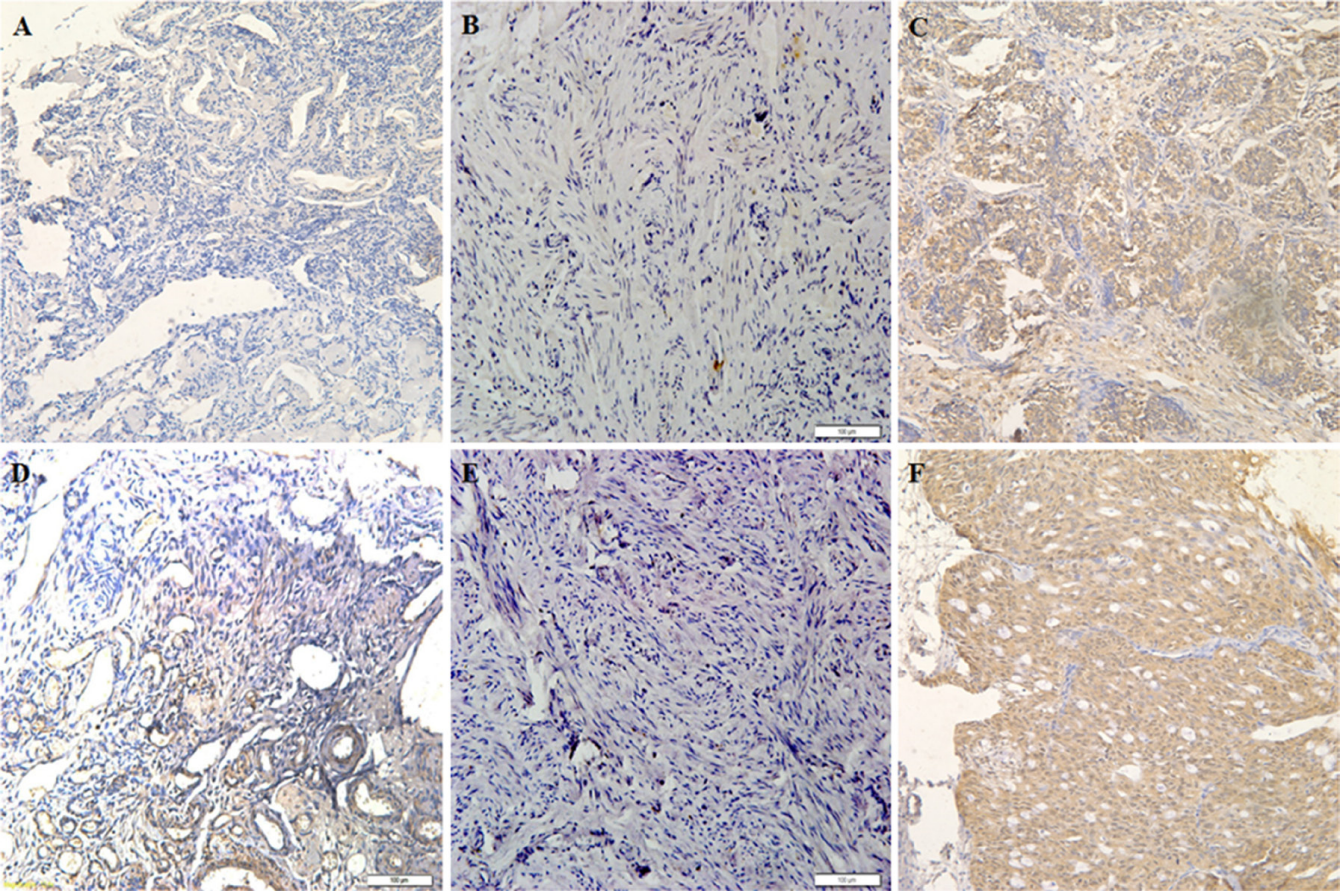
E2F-1 expression is correlated with pathogenesis and aggressiveness of ovarian carcinoma E2F-1 expression in normal ovarian tissue (**A** and **D**) oviduct tissues (**B** and **E**) and ovarian carcinoma tissues (**C** and **F**) which was detected by immunohistochemistry.

Immunohistochemical analysis of the clinical relevance of E2F1 expression in ovarian carcinoma showed that E2F1 expression was statistically significantly elevated in type I tumors (Table 1) compared to normal ovarian tissues (Figure 1A, 1D) and was positively associated with International Federation of Gynecology and Obstetrics (FIGO) stage I–II and III–IV disease. E2F1 expression was also significantly elevated in type II tumors (Table 2) compared to normal fallopian tube tissues (Figure 1B, 1E). The findings suggest that E2F1 has potent oncogenic effects in ovarian carcinoma (Figure 1C, 1F) of different type and origin.

**Table 1:** Relationship between E2F1 expression and clinicopathological features of Type I ovarian carcinomas

Clinicopathological feature	E2F1 (− = 0, + = 1, ++ = 2, +++ = 3)				Total	PR (%)	*P* value
	0	1	2	3			
**The pathology types**							
Normal ovarian tissues	16	1	0	0	17	5.88	**0.0027**
Type I tumors	32	21	6	0	59	45.76	
**FIGO stages**							**0.0005**
I–II	27	9	2	0	38	28.95	
III–IV	5	12	4	0	21	76.19	

PR = positive rate; Bold means *P* < 0.05.

Immunohistochemical analysis of the clinical relevance of E2F1 expression in ovarian carcinoma showed that E2F1 expression was statistically significantly elevated in type I tumors compared to normal ovarian tissues and was positively associated with International Federation of Gynecology and Obstetrics (FIGO) stage I–II and III–IV disease.

**Table 2:** Relationship between E2F1 expression and clinicopathological features of Type II ovarian carcinomas

Clinicopathological feature	E2F1 (− = 0, + = 1, ++ = 2, +++ = 3)				Total	PR (%)	*P* value
	0	1	2	3			
**The pathology types**							**0.0006**
Normal fallopian tubes	21	2	0	0	23	8.70	
Type II tumors	50	38	7	1	96	47.92	
**FIGO stages**							0.4717
II	8	9	1	0	18	55.56	
III–IV	42	29	6	1	78	46.15	

PR = positive rate; Bold means *P* < 0.05.

Immunohistochemical analysis of the clinical relevance of E2F1 expression in ovarian carcinoma showed that E2F1 expression was significantly elevated in type II tumors compared to normal fallopian tube tissues.

### Effects of E2F1 on ovarian carcinoma *in vitro* and *in vivo*

**Figure 2:**
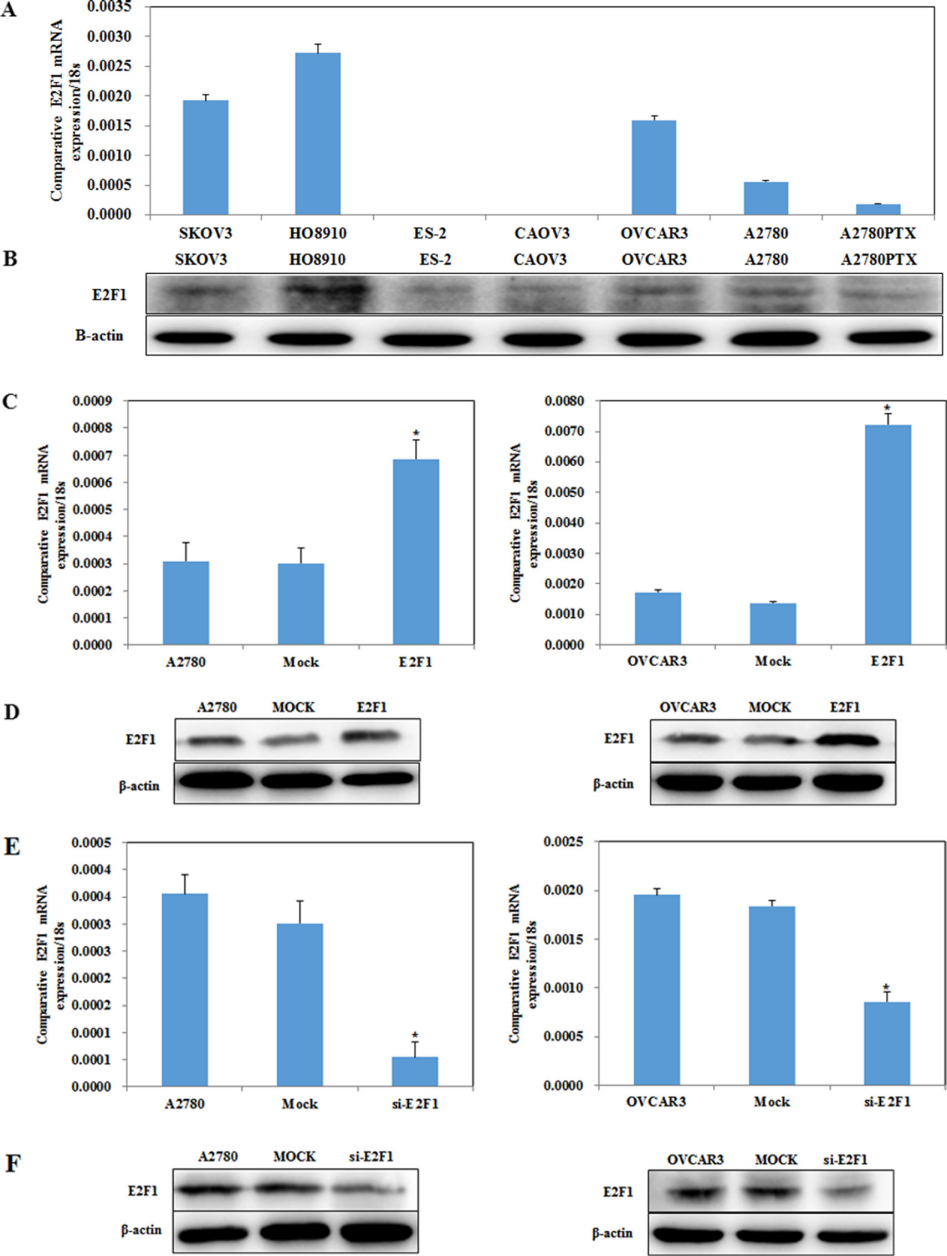
E2F1 expression in ovarian cancer cell lines E2F1 mRNA and protein expression levels among ovarian cancer cell lines SKOV3, HO8910, ES-2, CAOVR3, OVCAR3, A2780 and A2780PTX were examined (**A** and **B**). Transfection of A2780 and OVCAR3 cells with the E2F-1 plasmid results in increased E2F-1 (**C**) mRNA and (**D**) protein expression, while silencing of E2F-1 reduces (**E**) mRNA and (**F**) protein expression.

E2F1 mRNA and protein expression levels in SKOV3, HO8910, ES-2, CAOVR3, OVCAR3, A2780 and A2780PTX cells were examined by reverse transcription–PCR (RT-PCR) and western blotting (Figure 2A, 2B, *P* < 0.05), respectively. E2F1 plasmid and E2F1 short interfering RNA (siRNA) were transfected into the A2780 and OVCAR3 cell lines, which were selected for their intermediate *E2F1* expression, which allowed us to stably upregulate *E2F1* expression via E2F1 plasmid transfection (Figure 2C, 2D, *P* < 0.05) and to downregulate *E2F1* expression using the E2F1 siRNA (Figure 2E, 2F, *P* < 0.05). Following the transfection, we analyzed E2F1 levels by RT-PCR and western blot.

**Figure 3:**
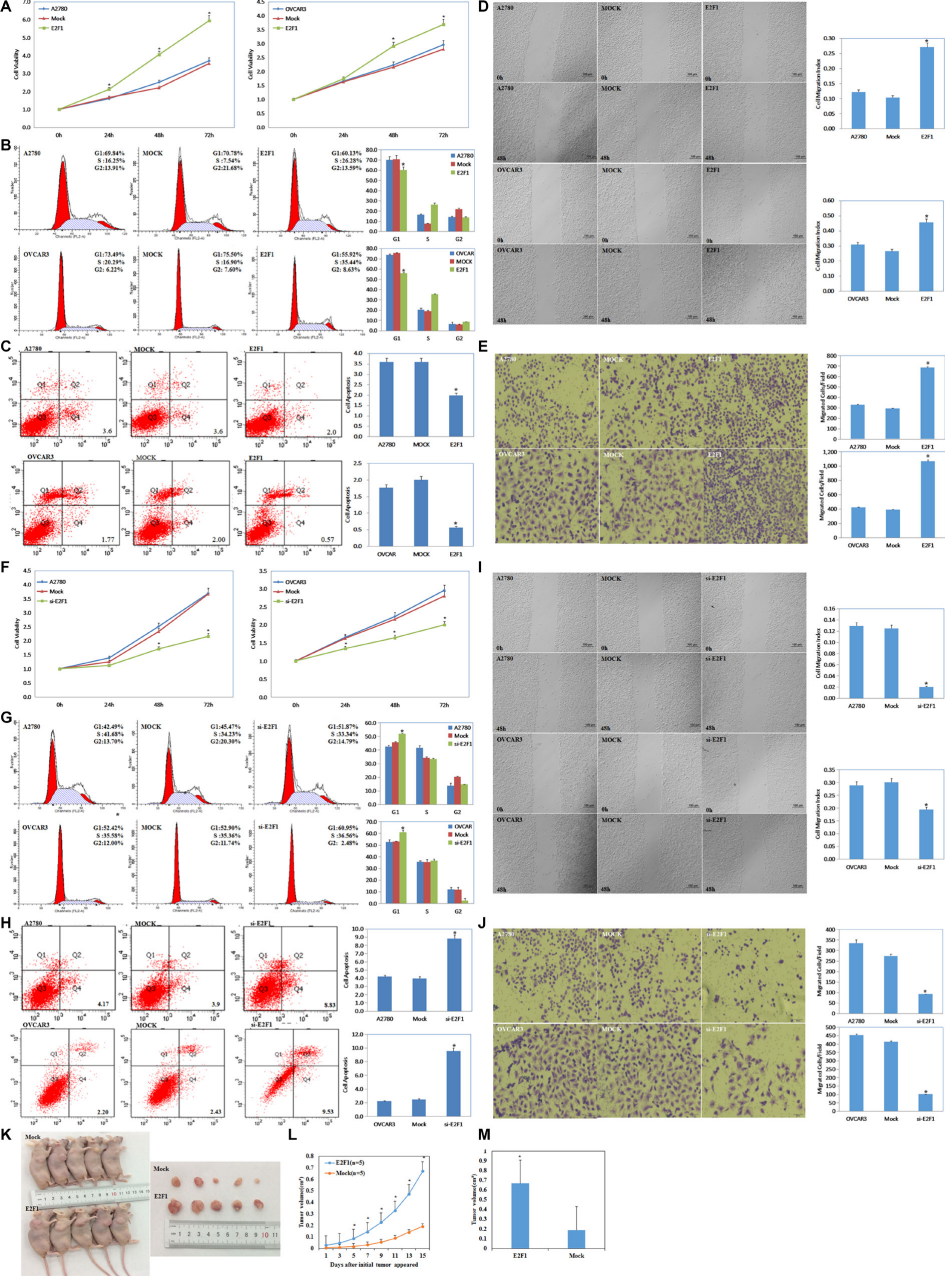
Effects of E2F1 on ovarian carcinoma *in vitro* and *in vivo* Overexpression of E2F-1 (**A**) increased the rate of growth, (**B**) promoted G1/S progression, (**C**) decreased levels of apoptosis, (**D**) increased migration and (**E**) invasion ability. Knockdown of E2F1 exhibit significantly (**F**) decreased growth, (**G**) increased G1 arrest and (**H**) increased apoptosis, (**I**) lower migration and (**J**) invasion ability. Nude mice injected with cells stably expressing E2F-1 showed a dramatic increase in tumor size (**K**) and tumor xenograft (**L**) growth and (**M**) volum *in vivo*. Results are representative of three separate experiments; data are expressed as the mean ± standard deviation. *p < 0.05.

The tetrazolium (MTT), cell cycle, apoptosis, wound healing, and cell invasion assays demonstrated that cells transfected with E2F1 plasmid had faster growth (Figure 3A, *P* < 0.05), promoted G1–S progression (Figure 3B, *P* < 0.05), decreased apoptosis (Figure 3C, *P* < 0.05), and increased migration (Figure 3D, *P* < 0.05) and invasive ability (Figure 3E, *P* < 0.05). In contrast, E2F1 siRNA caused severe growth retardation (Figure 3F, *P* < 0.05), increased the number of cells in G1 phase and reduced the number of cells in S phase (Figure 3G, *P* < 0.05), induced higher levels of apoptosis (Figure 3H, *P* < 0.05), and reduced cell migration (Figure 3I, *P* < 0.05) and invasive ability (Figure 3J, *P* < 0.05) as compared to the control and mock-transfected cells.

In the nude mouse xenograft model, mice injected with cells stably transfected E2F1 plasmid had a dramatic induction of tumor size (Figure 3K, *P* < 0.05) and faster tumor growth than the control mice (Figure 3L, 3M, *P* < 0.05), suggesting that E2F1 promotes tumorigenesis and growth.

### E2F1 targets the *miR-519d* promoter directly, and miR-519d targets *E2F1* mRNA directly

**Figure 4:**
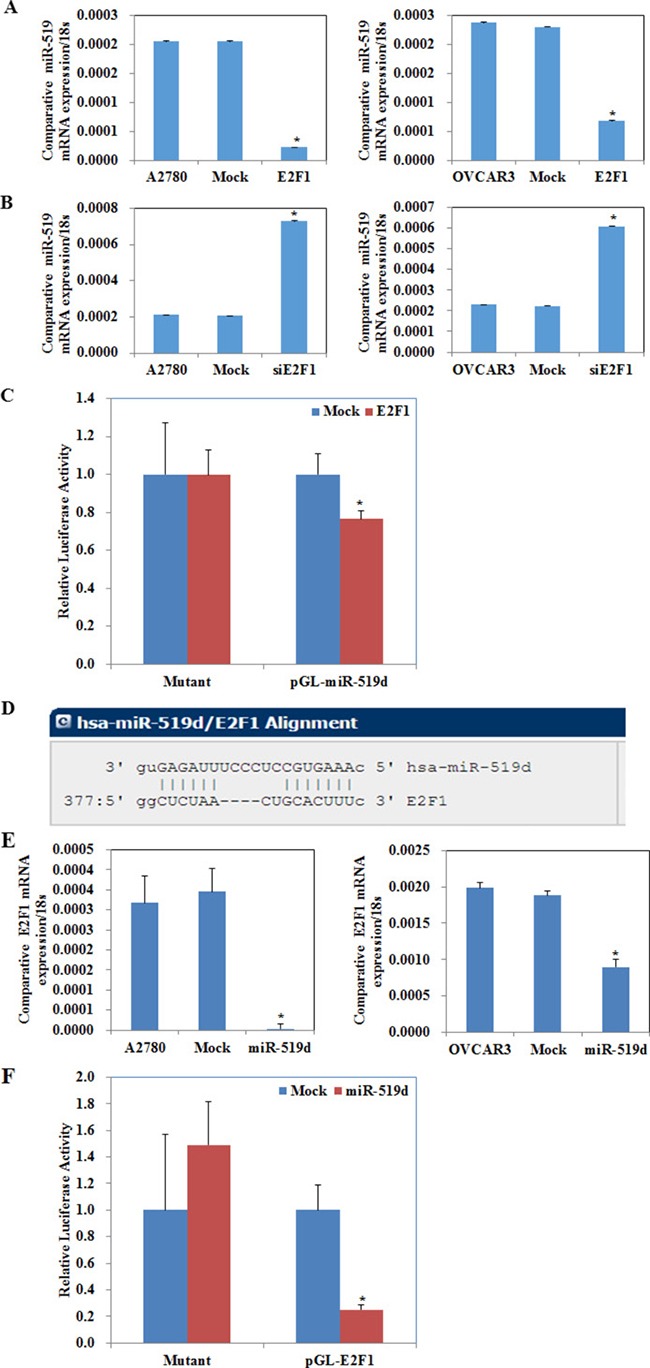
E2F1 downregulated miR-519d directly, and miR-519d downregulated E2F1 directly (**A**) Overexpression of E2F-1 represses miR-519d mRNA expression. (**B**) Silencing of E2F-1 promotes miR-519d mRNA expression. (**C**) Luciferase reporter assays confirm that miR-519d is the target of E2F-1. (**D**) The microRNA.org website predicted that the *E2F1* mRNA 3′ untranslated region (3′ UTR) contained miR-519d binding sites. (**E**) Overexpression of miR-519d represses E2F-1 mRNA expression. (**F**) Luciferase reporter assays confirm that E2F-1 is the target of miR-519d. *p < 0.05.

We searched target prediction websites (UCSC Genome Browser Home, http://genome.ucsc.edu/; JASPAR database, http://jaspar.genereg.net/) and predicted that the *miR-519d* promoter contains an E2F1 binding site. E2F1 transfection repressed *miR-519d* mRNA expression (Figure 4A, *P* < 0.05), while silencing E2F1 promoted it (Figure 4B, *P* < 0.05). The dual luciferase reporter assay showed that miR-519d–luciferase reporter plasmid and E2F1 plasmid co-transfection in HEK293 cells significantly reduced luciferase activity (Figure 4C, *P* < 0.05), with both mutant and control groups showing no significant changes in luciferase activity. These results demonstrate that E2F1 directly binds the *miR-519d* promoter to decrease miR-519d expression.

The microRNA.org website (http://microRNA.org) predicted that the *E2F1* mRNA 3′ untranslated region (3′ UTR) contained miR-519d binding sites (Figure 4D, *P* < 0.05). MiR-519d transfection downregulated *E2F1* mRNA expression (Figure 4E, *P* < 0.05), and there was fold reduction in the luciferase activity of HEK293 cells co-transfected with E2F1–luciferase reporter plasmid and miR-519d mimic when compared with the mutant and control groups (Figure 4F, *P* < 0.05), which suggests that miR-519d directly downregulates E2F1.

The above findings reveal that a direct repressive regulatory loop exists between E2F1 and miR-519d.

### MiR-519d inhibits ovarian carcinoma and downregulates RhoC directly

**Figure 5:**
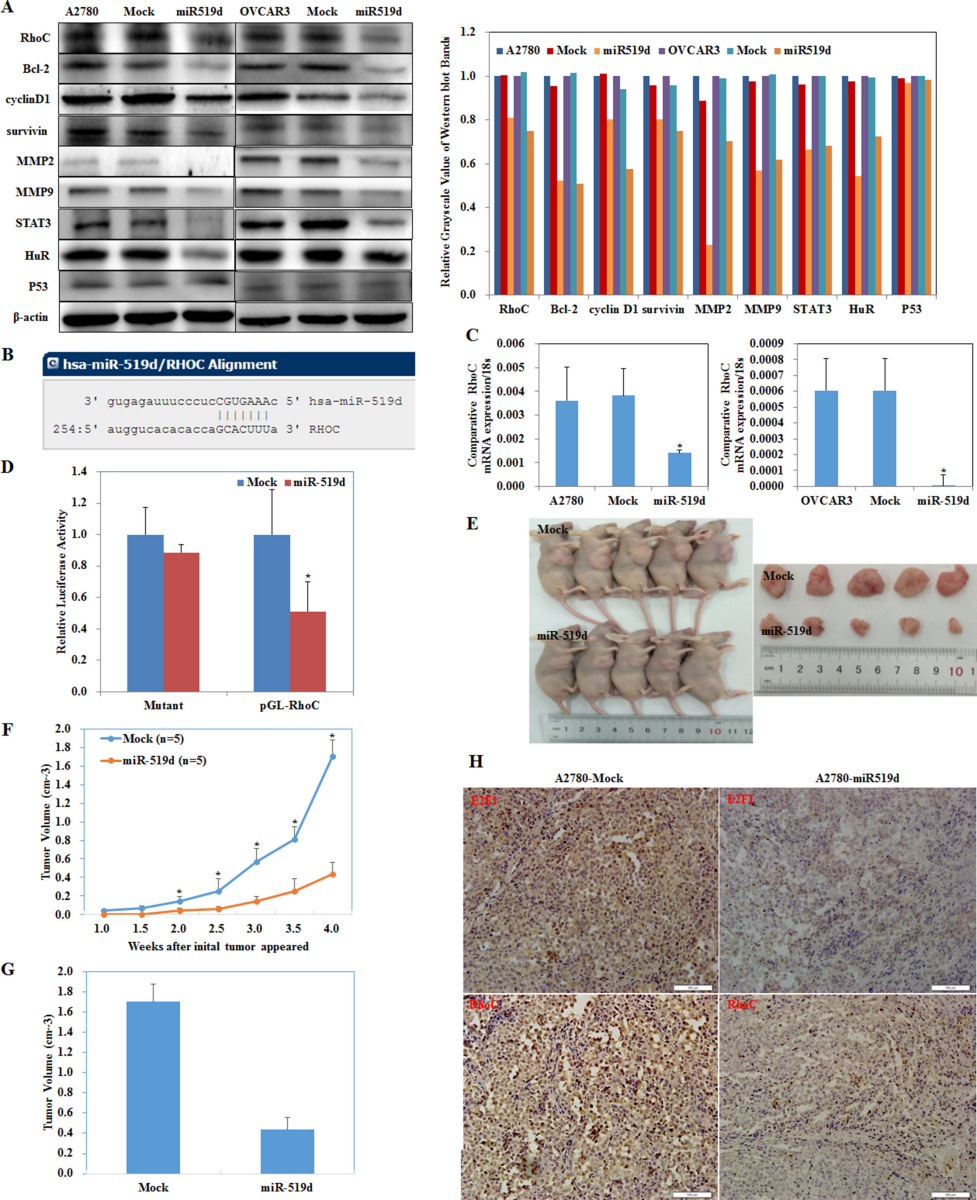
MiR-519d inhibits ovarian carcinoma and downregulates RhoC directly (**A**) miR-519d transfection in A2780 and OVCAR3 cells downregulated RhoC, Bcl-2, cyclin D1, survivin, MMP2, MMP9, STAT3, and HuR expression. No changes were observed in p53 expression. (**B**) The sequence in the 3′UTR of RhoC complementary to the miRNA-519d seed region suggests that RhoC is a direct target of miR-519d. (**C**) miR-519d transfection reduces mRNA expression of RhoC. (**D**) Dual luciferase reporter assay indicated that miR-519d directly targets RhoC by binding its 3′UTR. Nude mice injected with cells stably expressing miR-519d showed a (**E**) dramatic reduction in tumor size and tumor xenograft (**F**) growth and (**G**) volumn. (**H**) miR-519d overexpression downregulates E2F1 and RhoC expression in tumor xenografts *in vivo*. *p < 0.05.

Western blot showed that miR-519d transfection in ovarian carcinoma cells downregulated RhoC (Ras homolog gene family member C), Bcl-2, cyclin D1, survivin, MMP2, MMP9, STAT3 (signal transducer and activator of transcription 3), and HuR (ELAV-like RNA-binding protein 1) expression (Figure 5A, *P* < 0.05). No changes were observed in p53 expression. This suggests that miR-519d has suppressive effects on ovarian carcinoma by downregulating RhoC, Bcl-2, cyclin D1, survivin, MMP2, MMP9, STAT3 and HuR expression.

A target prediction website (http://microRNA.org) revealed that miR-519d binds the *RhoC* mRNA 3′ UTR region (Figure 5B). Transfecting miR-519d mimic into A2780 and OVCAR3 cell significantly decreased *RhoC* mRNA (Figure 5C, *P* < 0.05).

We generated a luciferase reporter gene containing the human *RhoC* mRNA 3′ UTR predicted to have potential miR-519d binding sites. Co-transfecting HEK293 cells with RhoC–luciferase reporter plasmid plus miR-519d mimic significantly reduced luciferase activity (Figure 5D, *P* < 0.05). Furthermore, luciferase activity was fully restored in the mutant and control groups, demonstrating that *RhoC* mRNA is a direct target of miR-519d.

The nude mouse xenograft model showed that mice injected with cells stably expressing miR-519d had obviously reduced tumor size (Figure 5E, *P* < 0.05) and tumor growth than the control mice (Figure 5F, 5G, *P* < 0.05). Immunohistochemistry showed that E2F1 and RhoC expression were downregulated in the tumor xenograft tissues of the miR-519d group as compared to the control (Figure 5H).

These results demonstrate that miR-519d directly decreases RhoC and downregulates the expression of Bcl-2, cyclin D1, survivin, MMP2, MMP9, STAT3, and HuR to suppress ovarian carcinoma.

### Effects of E2F1 on ovarian carcinoma cell genotype

**Figure 6:**
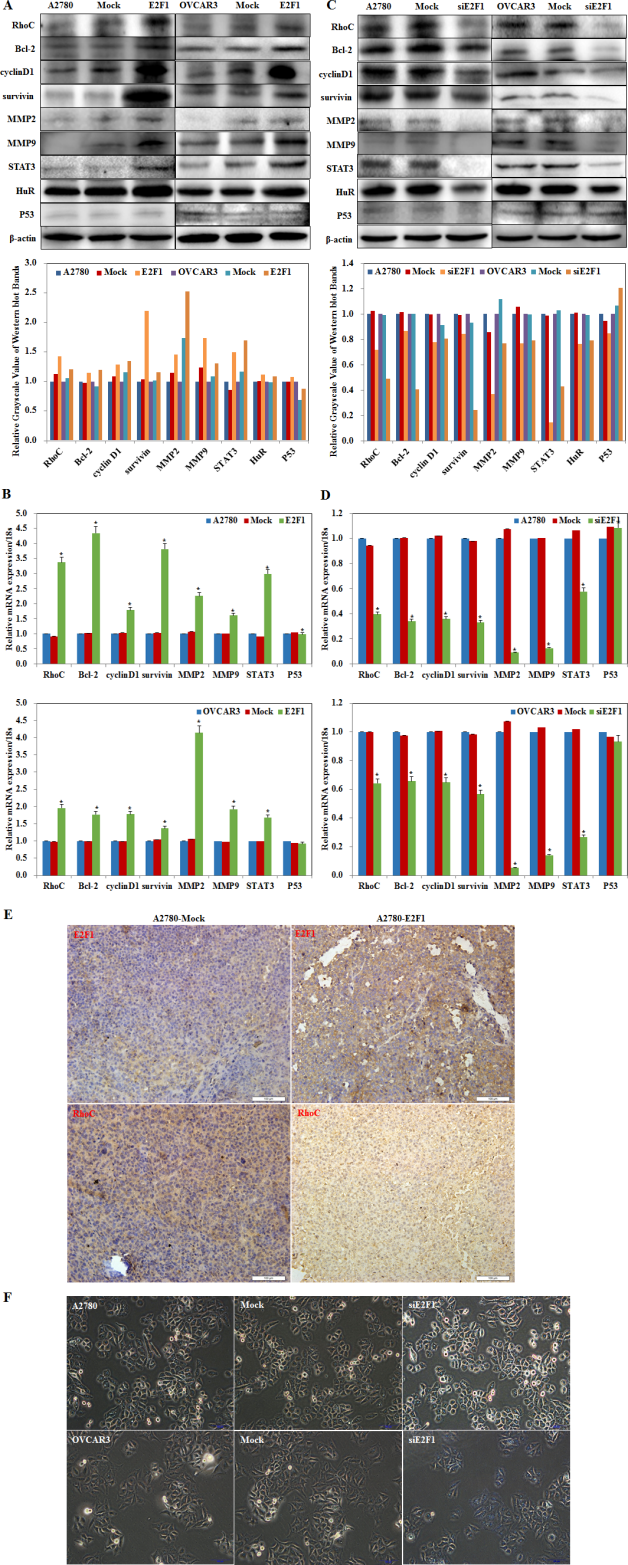
Effects of E2F1 on ovarian carcinoma cell genotype Overexpression of E2F-1 increases RhoC, Bcl-2, cyclin D1, survivin, MMP2, MMP9, STAT3, and HuR (**A**) protein and (**B**) mRNA expression. Silencing of E2F-1 decreases RhoC, Bcl-2, cyclin D1, survivin, MMP2, MMP9, STAT3, and HuR (**C**) protein and (**D**) mRNA expression. However, no significant differences were found in P53. (**E**) E2F-1–injected nude mice up-regulation of E2F-1 and RhoC expression in tumor xenografts. (**F**) silencing *E2F1* increased SA-β-gal (senescence-associate β-galactosidase) activity and triggered senescence. *p < 0.05

*E2F1* overexpression increased RhoC, Bcl-2, cyclin D1, survivin, MMP2, MMP9, STAT3, and HuR protein and mRNA expression (Figure 6A, 6B, *P* < 0.05); Silencing *E2F1* decreased RhoC, Bcl-2, cyclin D1, survivin, MMP2, MMP9, STAT3, and HuR protein and mRNA expression (Figure 6C, 6D, *P* < 0.05). No changes were observed in p53 expression.

The immunohistochemistry assay showed that E2F1 and RhoC expression were upregulated in the tumor xenograft tissue from nude mice of the E2F1 group when compared to the control group (Figure 6E, *P* < 0.05). The findings support the premise that E2F1 promotes ovarian carcinogenesis and growth by upregulating the expression of *RhoC* and Bcl-2, cyclin D1, survivin, MMP2, MMP9, STAT3, and HuR.

MiR-519d triggered cellular senescence by repressing HuR expression. The senescence detection kit showed that silencing *E2F1* increased SA-β-gal (senescence-associate β-galactosidase) activity and triggered senescence as compared to the control groups (Figure 6F, *P* < 0.05). The data indicate that silencing *E2F1* directly targeted miR-519d to downregulate HuR, inducing cellular senescence.

### Effects of E2F1 and miR-519d co-transfection on ovarian carcinoma cells

**Figure 7:**
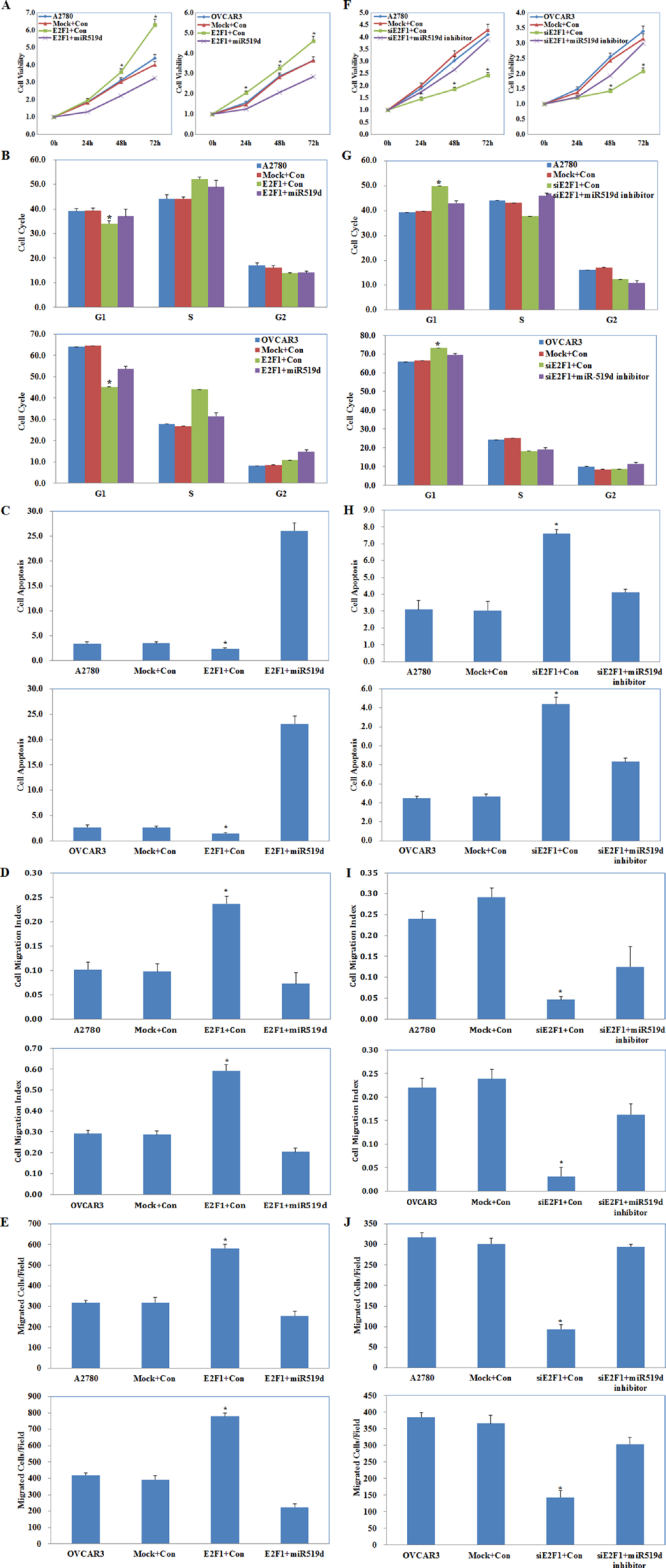
Effects of E2F1 and miR-519d co-transfection on ovarian carcinoma cells E2F1 plasmid and miR-519d mimic co-transfection (Mock+Con stands for E2F1-mutant plus miR-519d contorl, E2F1+Con stands for E2F1 plasmid plus miR-519d control ) caused (**A**) severe growth retardation, (**B**) promoting G1–S progression, (**C**) high levels of apoptosis, and reduced (**D**) migration and (**E**) invasive ability. E2F1 siRNA and miR-519d inhibitor co-transfection (Mock+Con stands for siE2F1-mutant plus miR-519d inhibitor contorl, siE2F1+Con stands for E2F1 siRNA plus miR-519d inhibitor control ) exhibited (**F**) slower growth, (**G**) G1 arrest, (**H**) increased apoptosis, (**I**) decreased migration and (**J**) invasive ability. *p < 0.05. See Supplementary Figure 1.

A2780 and OVCAR3 cells were co-transfected with E2F1 plasmid and miR-519d mimic or with E2F1 siRNA and miR-519d inhibitor. The MTT, cell cycle, apoptosis, wound healing, and cell invasion assays showed that E2F1 plasmid and miR-519d mimic co-transfection reversed the effect of E2F1 alone, causing severe growth retardation (Figure 7A, *P* < 0.05), high levels of apoptosis (Figure 7C, *P* < 0.05), and reduced migration (Figure 7D, *P* < 0.05) and invasive ability (Figure 7E, *P* < 0.05) as compared to the control and mock cells, but had the same effect as E2F1 in promoting G1–S progression (Figure 7B, *P* < 0.05). E2F1 siRNA and miR-519d inhibitor co-transfection partly counteracted the effects of silencing *E2F1* on cell proliferation, the cell cycle, apoptosis, migration, and invasion, although the cells still exhibited slower growth (Figure 7F, *P* < 0.05), G1 arrest (Figure 7G, *P* < 0.05), increased apoptosis (Figure 7H, *P* < 0.05), decreased migration (Figure 7I, *P* < 0.05) and invasive ability (Figure 7J, *P* < 0.05) as compared with the control and mock cells.

These results demonstrate that miR-519d plays a central role in E2F1 promotion of ovarian carcinoma cell proliferation, apoptosis, and cell migration and invasion, but not on the cell cycle.

### Effects of E2F1 and RhoC co-transfection on ovarian carcinoma cells

**Figure 8:**
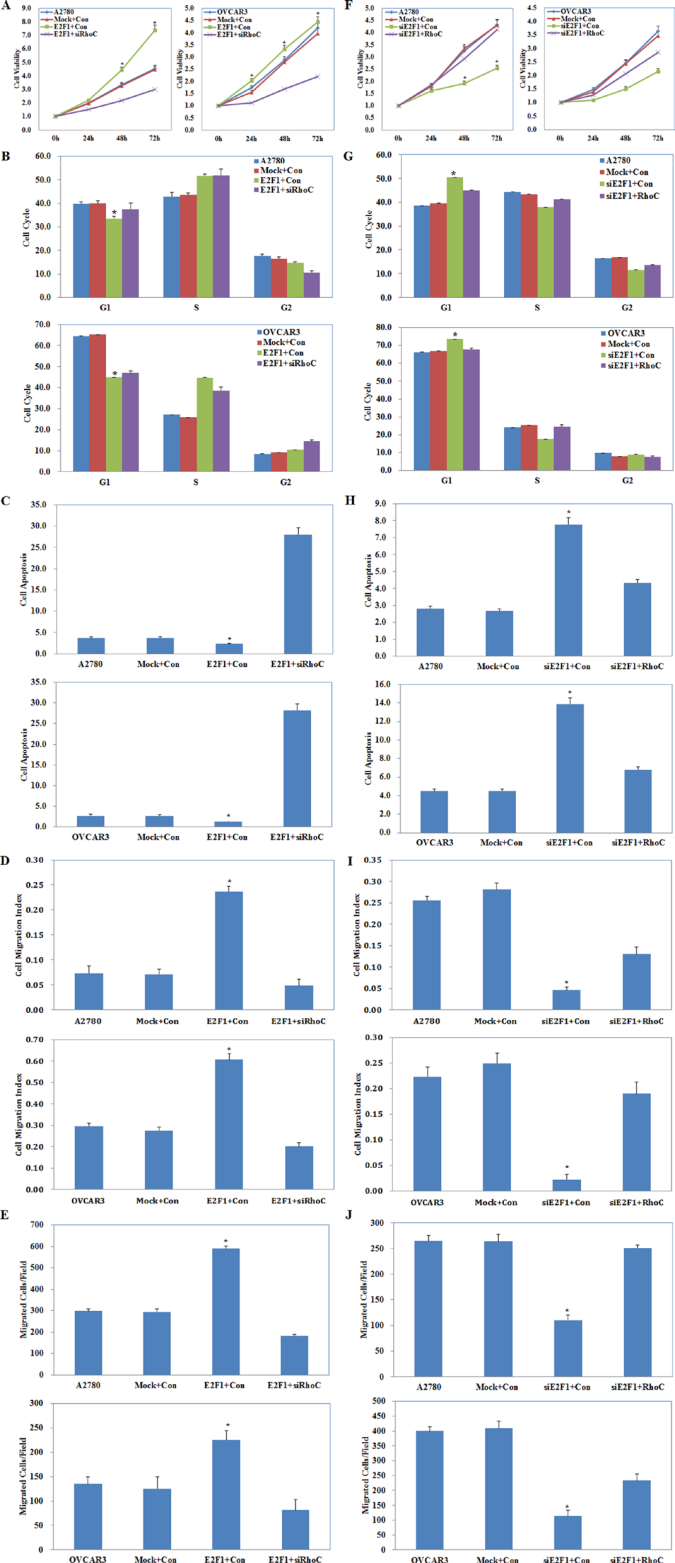
Effects of E2F1 and RhoC co-transfection on ovarian carcinoma cells E2F1 plasmid and RhoC siRNA co-transfection (Mock+Con stands for E2F1-mutant plus siRhoC contorl, E2F1+Con stands for E2F1 plasmid plus siRhoC control ) caused (**A**) severe growth retardation, (**B**) promoting G1–S progression, (**C**) higher levels of apoptosis, and (**D**) reduced cell migration and (**E**) invasive ability. E2F1 siRNA and RhoC plasmid co-transfection (Mock+Con stands for siE2F1-mutant plus RhoC contorl, siE2F1+Con stands for E2F1 siRNA plus RhoC control ) exhibited (**F**) slower growth, (**G**) G1 arrest, (**H**) increased apoptosis, and decreased (**I**) migration and (**J**) invasive ability as compared to the control and mock cells. *p < 0.05. See Supplementary Figure 2.

A2780 and OVCAR3 cells were co-transfected with E2F1 plasmid and RhoC siRNA or with E2F1 siRNA and RhoC plasmid. Cell functional assays were performed to analyze the effects on A2780 and OVCAR3 cell proliferation, cycle, apoptosis, migration, and invasion. E2F1 plasmid and RhoC siRNA co-transfection reversed the effect of E2F1 alone, causing severe growth retardation (Figure 8A, *P* < 0.05), higher levels of apoptosis (Figure 8C, *P* < 0.05), and reduced cell migration (Figure 8D, *P* < 0.05) and invasive ability (Figure 8E, *P* < 0.05), but had the same effect in promoting G1–S progression (Figure 8B, *P* < 0.05) when compared with the control and mock cells. E2F1 siRNA and RhoC plasmid co-transfection partly counteracted the effects of *E2F1* silencing on cell proliferation, the cell cycle, apoptosis, and cell migration and invasion, although the cells still exhibited slower growth (Figure 8F, *P* < 0.05), G1 arrest (Figure 8G, *P* < 0.05), increased apoptosis (Figure 8H, *P* < 0.05), and decreased migration (Figure 8I, *P* < 0.05) and invasive ability (Figure 8J, *P* < 0.05) when compared with the control and mock cells.

These results demonstrate that RhoC plays a central role in the oncogenic regulation by E2F1/miR-519d on ovarian carcinoma cell proliferation, apoptosis, migration, and invasion, but not on the cell cycle.

## DISCUSSION

According to the most recent National Comprehensive Cancer Network (NCCN) guidelines, epithelial ovarian cancer is grouped into type I ovarian cancer (mostly originating from the ovary) and type II ovarian cancer (mostly originating from the oviduct) [31]. We compared *E2F1* expression levels in 59 cases of type I tumors and 17 normal ovarian tissues, and *E2F1* expression was significantly elevated in the ovarian carcinoma tissues and was positively associated with FIGO stage I–II and III–IV disease. We also compared *E2F1* expression levels in 96 cases of type II tumors and 23 fallopian tube tissues, and *E2F1* expression was also significantly elevated in the ovarian carcinoma tissues. The finding is consistent with the study by Suh and colleagues [27]. We found that E2F1 promoted ovarian carcinoma cell proliferation, G1–S progression, survival, migration, and invasion *in vitro* and promoted ovarian carcinoma tumorigenesis and tumor growth *in vivo*. We also observed that E2F1 increased Bcl-2, cyclin D1, survivin, MMP2, and MMP9 expression in ovarian carcinoma, but did not alter p53 expression. These results are similar to the oncogenic role of E2F1 in small cell lung cancer [28] and clear cell renal cell carcinoma [29], but differ from the suppressive effect of E2F1 in gastric cancer [17–19] and Hodgkin lymphoma [25]. Our findings suggest that *E2F1* functions as an oncogene in ovarian carcinoma in part by upregulating Bcl-2, cyclin D1, survivin, MMP2, and MMP9 expression.

To explore the underlying molecular mechanism of the oncogenic effects of E2F1 on ovarian carcinoma, we focused on microRNAs (miRNAs). MiRNAs are small, non-coding, single-stranded, and evolutionarily conserved RNAs that regulate gene expression by binding directly to complementary sites in the 3′ UTR of target mRNAs; the process plays a crucial role in tumor development and progression [32–33]. Previous studies have proved that transcription factors act together with miRNAs in regulating the target genes [34].

The dual luciferase reporter assay confirmed the prediction that the *miR-519d* promoter sequence contains an E2F1 binding site. E2F1 overexpression suppressed *miR-519d* mRNA expression, proving that E2F1 targets the *miR-519d* promoter directly to decrease its expression. MiR-519d belongs to the chromosome 19 miRNA cluster (C19MC), and acts as a tumor suppressor in breast cancer, chondrosarcoma, osteosarcoma, and hepatocellular carcinoma [35–38], and it is significantly downregulated in ovarian cancer cell lines and tissues [39]. MiR-519d decreased ovarian cancer cell proliferation and sensitized ovarian cancer cells to cisplatin-induced cell death [40]. We conclude that miR-519d has the same suppressive effects on ovarian carcinoma based on the significantly decreased tumor size and growth in the nude mouse xenograft model. MiR-519d suppresses trophoblast cell invasion and migration by downregulating MMP2 [41], suppresses breast cancer by inhibiting STAT3 expression [35], and inhibits tumor metastasis and MMP2 expression in chondrosarcoma and osteosarcoma [36–37]. MiR-519d reduces cell proliferation and inhibits tumorigenesis largely by decreasing the levels of the RNA-binding protein HuR to trigger senescence in ovarian carcinoma cells [42–44]. We found that miR-519d transfection decreased *MMP2*, *MMP9*, *STAT3*, and *HuR* expression in ovarian carcinoma cells; at the same time, E2F1 increased the expression of these genes. We found that silencing *E2F1* triggered senescence in ovarian carcinoma cells, supporting the premise that E2F1 targets *miR-519d* directly, decreasing its expression. We co-transfected ovarian carcinoma cells with E2F1 plasmid and miR-519d mimic, and found that miR-519d greatly reversed the effect of E2F1, revealing that miR-519d plays a central role in the oncogenic effects of E2F1 on ovarian carcinogenesis and development.

We found that RhoC is a putative and direct target of miR-519d, which the dual luciferase reporter assay confirmed. We observed that miR-519d transfection decreased RhoC expression and that *E2F1* overexpression increased RhoC expression in ovarian carcinoma cell lines; RhoC expression was decreased in miR-519d tumor xenograft tissue and was increased in E2F1 tumor xenograft tissue when compared with their respective controls. RhoC is a small G protein/guanosine triphosphatase closely involved in tumor invasion and metastasis [45–47], and could serve as a good biomarker of ovarian carcinoma differentiation and progression [48–49]. We found that E2F1 plasmid and RhoC siRNA co-transfection greatly reversed the oncogenic role of E2F1 in ovarian carcinoma cell phenotype, revealing that RhoC plays a central role in the oncogenic regulation of E2F1/miR-519d on ovarian carcinogenesis and development. The above findings demonstrate that E2F1 promotes ovarian carcinoma by directly targeting miR-519d, suppressing it, which in turn targets RhoC expression, downregulating it. Conversely, miR-519d mimic decreased *E2F1* expression. The prediction that miR-519d would target the *E2F1* mRNA 3′ UTR was in agreement with the results of the dual luciferase reporter assay. Furthermore, *E2F1* expression was lower in the miR-519d xenograft group, proving that miR-519d also targets *E2F1* to downregulate its expression. These findings indicate an E2F1–miR-519d direct repressive regulatory loop.

In the E2F family of transcription factors, E2F2, E2F3, E2F4, and E2F8 expression is increased and may play oncogenic roles in ovarian carcinoma [26, 30, 50–51], and exert the same effects as E2F1. We searched target prediction websites and found that in addition to E2F1, the *miR-519d* promoter also contains E2F4 binding sites. Taken together, we hypothesize that E2F4 could have the same oncogenic effects as E2F1 on ovarian carcinoma by suppressing miR-519d. However, further research is needed to confirm this.

In conclusion, our study identifies a novel molecular mechanism for E2F1, whereby it targets miR-519d to upregulate RhoC, Bcl-2, cyclin D1, survivin, MMP2, MMP9, STAT3 and HuR expression, promoting tumorigenesis and progression in ovarian carcinoma. The E2F1/miR-519d/RhoC signaling pathway is of significant importance and shows promise as a target for diagnosing and treating ovarian carcinoma.

## MATERIALS AND METHODS

### Ovarian carcinoma specimens

According to the latest NCCN guidelines, ovarian cancer were grouped into type I ovarian cancer (most origin from ovaries) and type II ovarian cancer (most origin from oviducts). Consisting of type I tumors 59 cases and type II tumors 96 cases, 17 normal ovarian tissues cases and 23 fallopian tube tissues cases were collected from patients undergoing surgical resection at the Department of Gynecology of the First Affiliated Hospital of China Medical University (Shenyang, Liaoning, China) between 2003 and 2014. The tumor specimens were microscopically confirmed by pathologists. None of the patients had received preoperative chemotherapy or radiotherapy. Informed consent was obtained from all subjects, the study was approved by the China Medical University Ethics Committee, and all specimens were handled and made anonymous according to ethical and legal standards. The average age at surgery was 54.3 years (range, 18–76 years). Each ovarian carcinoma specimen was evaluated according to the 2014 FIGO staging system. The histological architecture of ovarian carcinoma was defined in terms of World Health Organization classification.

### Cell culture and transfection

The ovarian carcinoma cell lines OVCAR3 (serous cystic adenocarcinoma) and A2780 (serous cystic adenocarcinoma) were cultured in RPMI 1640 (HyClone, Logan, USA), while A2780 cells were cultured in DMEM (HyClone). Both mediums were supplemented with 10% fetal bovine serum (FBS), 100 U/mL penicillin, and 100 μg/mL streptomycin. All cell lines were maintained at a temperature of 37°C and a humidified atmosphere of 5% CO2. The medium was changed every two days for optimized culture conditions. Transfections were performed using Lipofectamine 2000 transfection reagent according to the manufacture's protocol. The sequence of siRNA targeting E2F-1 was: 5′-CGCUAUGAGACCUCACUG-3′. The sequence of the miR-519d mimic was: 5′-CAAAGTGCCTCCCTTTAGAGTG-3′. The target sequences of the RhoC siRNA were 5′-GUGCCUUUGG CUACCUUGAdTdT-3′ (sense) and 5′-UCAAGGUA GCCA AAGGCACdTdT-3′ (anti-sense).

### MTT assay

Ninety-six well plates were seeded with 3 × 103 cells per well. At designated time points following transfection (0 h, 24 h, 48 h, and 72 h), 20 μL of 5 mg/mL MTT (Sigma,USA) was added into every well and cells incubated at 37°C for 4 hours. The liquid was then removed and l50 μL of dimethylsulfoxide (DMSO, Sigma, USA) added to dissolve the precipitated formazan. Following shaking for 10 min, the optical density (OD) was measured at 490 nm on a microplate spectrophotometer (Bio-Tek Instruments, Winooski, USA).

### Cell cycle analysis

More than 1 × 104 cells were trypsinized, collected, washed twice with phosphate buffered saline (PBS), and fixed in 70% ethanol at −20°C for at least 12 h. The cells were washed twice with PBS and incubated with 400 μL RNase (0.25 mg/mL) at 37°C for 1 h, then resuspended in 100 μL Propidium Iodide(PI, KeyGen, NanJing China) and incubated at 4°C in the dark for 30 min. The PI cell cycle profile was detected by flow cytometry.

### Cell apoptosis assay

Cells were harvested at 1500 rpm for 5 min and washed twice with cold PBS. They were then resuspended in a mix of 50 μL binding buffer, 5 μL annexin V-FITC and 5 μL PI (KeyGen, China). Samples were gently vortexed and incubated in the dark for 15 min at room temperature. Two hundred microliters of binding buffer was added to each tube and samples were examined by flow cytometer within 1 h.

### Wound healing assay

Cells were seeded into 6-well plates and cultured for approximately 24 h to reach 80% confluence in the presence of mitomycin C(10 ug/ml, Sigma, USA). The monolayer was then scratched with a 200 μL pipette tip to create a wound. The 6-well plates were washed twice with PBS, and cells cultured in FBS-free medium. Cells were transfected with the miRNA or siRNA (or controls) at the same time. Prior to the wound healing assay, the width of each wound was examined by microscope and photographed using Image J software (National Institutes of Health, Bethesda, USA) at 0 h, 24 h and 48 h after wounding. Cell migration was measured by subtracting the wound width at 24 h or 48 h from that at 0 h.

### Cell invasion assay

Cell invasion assays were performed using Transwell chambers (24-well inserts; 8 μm-pore size; BD Bioscience, San Jose, CA, USA) pre-coated with 40 μL of Matrigel basement membrane matrix (Matrigel at a dilution of 1:10) incubated for 4 h at 37°C in 5% CO2. Two hundred microliters of serum-free medium containing 5 × 104 cells/well of transfected or non-transfected cells were placed in the upper chamber in the presence of mitomycin C (10 ug/ml, Sigma, USA), and the lower chambers filled with 600 μL of the same complete medium. The plates were incumanuscripting cells at the bottom of the upper chamber were washed twice with PBS. Cells on the underside of the filters were examined and counted using an Olympus fluorescence microscope (Tokyo, Japan). Cells were counted three times, and each experiment was repeated in triplicate.

### Cell senescence

Cells were washed once with PBS, 1 ml of β-galactosidase staining fixative was added and fixed at room temperature for 15 minutes, Then cells were washed three times with PBS for 3 minutes each, after that 1 ml of working solution was added to each well, incubated overnight at 37°C, then observed cells under ordinary light microscope.

### Real-time RT-PCR

Total RNA extraction from ovarian carcinoma cell lines and ovarian tissues was performed with 1 mL TRIzol reagent (Takara, Shiga, Japan) and the total RNA was reverse-transcribed to complementary DNA (cDNA) using the avian myeloblastosis virus reverse transcriptase and random primers (Takara, Shiga, Japan) according to the manufacturer's instructions. Real-time PCR amplification of the cDNA was performed in 20 μL reactions according to the SYBR Premix Ex Taq II kit (Takara, Shiga, Japan) protocol; 18S rRNA was used as the internal control. Hairpin-it microRNA Normalization RT-PCR Quantitation (GenePharma) was used to check E2F-1. The oligonucleotide primers for PCR were based on GenBank sequences. The primers used for E2F-1 and GAPDH were as follows: E2F-1 forward, 5-CCATCCAGGAAAAGGTGTGAA-3 and reverse, 5-AGCGCTTGGTGGTCAGATTC-3′.

### Western blotting

Cells were harvested and lysed with ice-cold lysis buffer (Sigma, USA) and protein concentration determined using a protein assay kit (Bio-Rad Laboratories, Hercules, USA). Denatured proteins (100 μg) were separated on 10% sodium dodecyl sulfate (SDS)-polyacrylamide gels, transferred to Hybond membranes (Amersham, Munich, Germany), and blocked overnight in 5% skimmed milk in Tris-buffered saline with Tween 20 (TBST). For immunoblotting, the membrane was incubated with antibodies against E2F-1, RhoC (1:300, Santa Cruz Biotechnology, Santa Cruz, USA), p53, caspase 3, stat3, Bcl-2, cyclin D1, matrix metalloproteinase (MMP) 2 and MMP9 (1:300, Bioss,BeiJing, China), HuR(proteintech, Chicago, USA). The membranes were then rinsed with TBST and incubated with anti-mouse or anti-rabbit IgG antibodies conjugated to horseradish peroxidase (1:5000; Dako, Carpinteria, USA) for 2 h. Bands were visualized on X-ray film (Fuji film, Tokyo, Japan) using Image Quant LAS 4000 (Fuji film) and ECL Plus detection reagents (Santa Cruz Biotechnology). β-actin (ZSGB-Bio,BeiJing, China) was used as a loading control.

### Dual luciferase reporter assay

The plasmid were designated pGL3-RhoC, pGL3-miR-519d and pGL3-E2F1. The pGL3-RhoC mutant plasmid, pGL3-miR-519d mutant plasmid and pGL3-E2F1 mutant plasmid containing nucleotide mutations in the predicted binding site based on the the website searching in GenBank and inserted into the downstream region of the firefly luciferase reporter (Promega, Madison, WI, USA). HEK293T Cells were seeded at 1 × 105 cells per well in 24-well plates, and cultured until 70% confluent. Cells were then co-transfected with the the pGL3-plasmid and relate plasmid or mimic (50 nM, Ribobio Biotechnology); pGL3-mutant, plasmid-mutant and control (400 ng, Promega) using Lipofectamine 2000. After 48 hours cells were lysed using cell lysis buffer (Cell Signaling, Boston, USA) and luciferase activity was measured using the Dual Luciferase Reporter Assay System (Promega BioSciences, San Luis Obispo, USA) according to the manufacturer's instructions. The luciferase activities were normalized to that of Renilla luciferase. The results were expressed as the means ± SD of at least three independent experiments.

### *In vivo* xenografts

Female 4–5 week-old, 18~20 g BALB/c nude mice were obtained from Vital River Laboratories (Beijing, China) and housed in a SPF grade environment. Subcutaneous tumor xenografts were established via injection of 1 × 10^7^ A2780 cells suspended in 200 μL PBS with (E2F-1 group or miR-519d group) or without (mock group) LV-hsa-E2F-1 transfection or LV-hsa-miR-519d transfection (Genechem, Shanghai, China) directly hypodermic in. All animal research protocols were performed following the National Institutes of Health Guide for the Care and Use of Laboratory Animals and were approved by the China Medical University Animal Care and Use Committee.

### Immunohistochemistry

Consecutive tissue sections were deparaffinized with xylene, rehydrated with alcohol, and subjected to antigen retrieval by heating in target retrieval solution (Dako) for 15 min in a microwave oven (Oriental Rotor). The sections were quenched with 3% hydrogen peroxide for 20 min to block endogenous peroxidase activity. Non-specific binding was prevented by adding 5% bovine serum albumin for 5 min. The sections were incubated at 4°C overnight with anti-E2F1 or anti-RhoC antibodies, and then incubated with HRP-conjugated anti-rabbit antibodies (Dako) for 2 h. After each treatment, the slides were washed three times with TBST for 5 min, and the binding sites were visualized with 3, 3′-diaminobenzidine. After counterstaining with Mayer's hematoxylin, the sections were dehydrated, cleared and mounted. Negative controls were prepared by omitting the primary antibody.

### Statistical analysis

Statistical evaluation was performed using the Spearman correlation test to analyze the rank data and the Mann-Whitney *U* test to differentiate the means of different groups. Cox's proportional hazards model was employed for multivariate analysis. A *p-value* of < 0.05 was considered statistically significant. SPSS 17.0 (SPSS, Chicago, IL, USA) software was employed to analyze all data.

## SUPPLEMENTARY MATERIALS FIGURES


