# Supplementary material for: E2F-1 targets miR-519d to regulate the expression of the ras homolog gene family member C
Source: Oncotarget. 2017 Jan 27;8(9):14777–93. doi: 10.18632/oncotarget.14833 (PMC5362443; doi:10.18632/oncotarget.14833)
Supplement: Supplementary file 1 [file oncotarget-08-14777-s001.pdf]

E2F-1 targets miR-519d to regulate the expression of the ras homolog gene family member C

Supplementary Materials

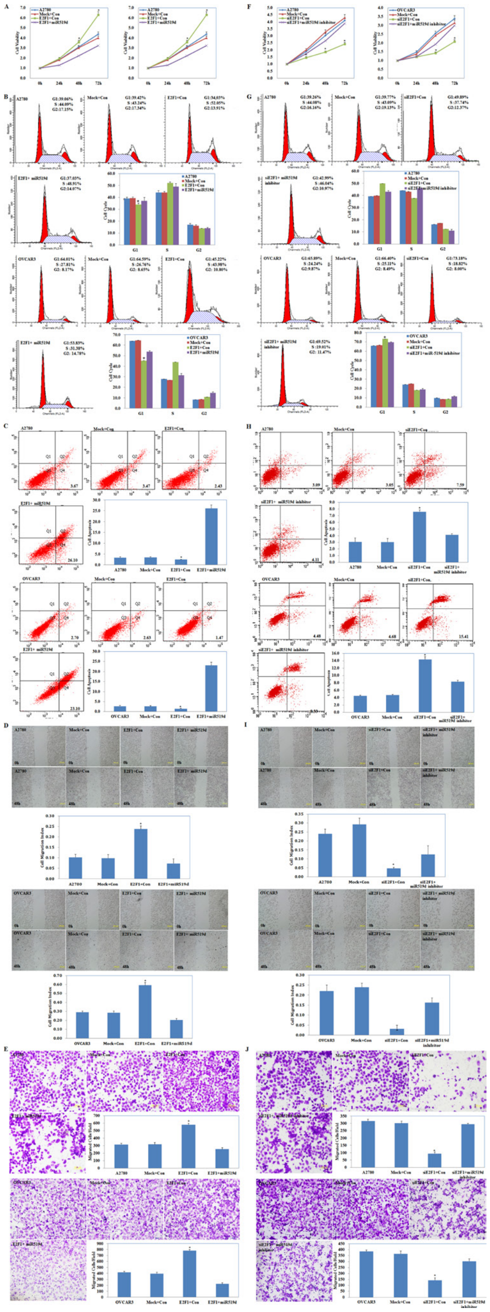

Supplementary Figure 1: Effects of E2F1 and miR-519d co-transfection on ovarian carcinoma cells. E2F1 plasmid and miR-519d mimic co-transfection (Mock+Con stands for E2F1-mutant plus miR-519d contorl, E2F1+Con stands for E2F1 plasmid plus miR-519d control) caused (**A**) severe growth retardation, (**B**) promoting G1–S progression, (**C**) high levels of apoptosis, and reduced (**D**) migration and (**E**) invasive ability. E2F1 siRNA and miR-519d inhibitor co-transfection (Mock+Con stands for siE2F1-mutant plus miR-519d inhibitor contorl, siE2F1+Con stands for E2F1 siRNA plus miR-519d inhibitor control) exhibited (**F**) slower growth, (**G**) G1 arrest, (**H**) increased apoptosis, (**I**) decreased migration and (**J**) invasive ability. *$p < 0.05$

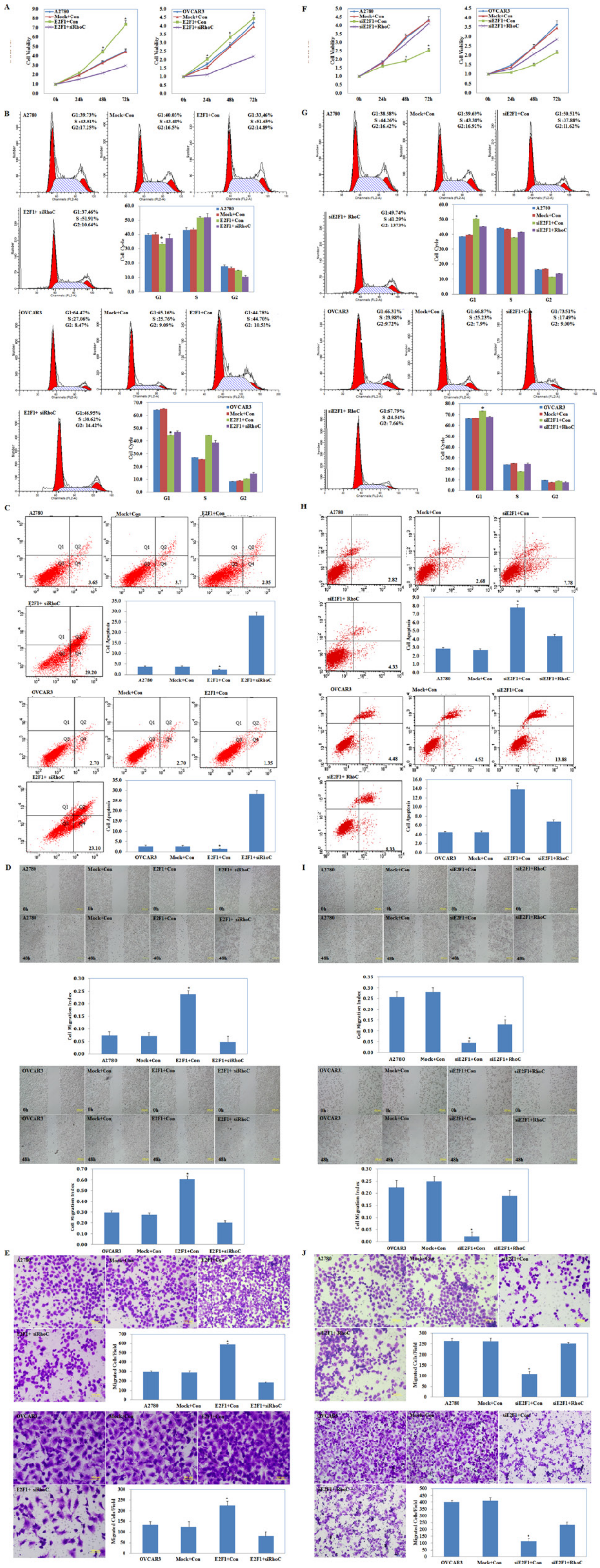

Supplementary Figure 2: Effects of E2F1 and RhoC co-transfection on ovarian carcinoma cells. E2F1 plasmid and RhoC siRNA co-transfection (Mock+Con stands for E2F1-mutant plus siRhoC contorl, E2F1+Con stands for E2F1 plasmid plus siRhoC control) caused (**A**) severe growth retardation, (**B**) promoting G1–S progression, (**C**) higher levels of apoptosis, and (**D**) reduced cell migration and (**E**) invasive ability. E2F1 siRNA and RhoC plasmid co-transfection (Mock+Con stands for siE2F1-mutant plus RhoC contorl, siE2F1+Con stands for E2F1 siRNA plus RhoC control) exhibited (**F**) slower growth, (**G**) G1 arrest, (**H**) increased apoptosis, and decreased (**I**) migration and (**J**) invasive ability as compared to the control and mock cells. *$p < 0.05$
